# Non-adjustable gravitational valves or adjustable valves in the treatment of hydrocephalus after aneurysmal subarachnoid hemorrhage patients?

**DOI:** 10.1007/s00701-022-05361-0

**Published:** 2022-09-23

**Authors:** Sebastian Arts, Jasper Hans van Lieshout, Martine van Bilsen, Cihat Karadag, Thomas Beez, Leonie van den Abbeele, Rene Aquarius, Saman Vinke, Ronald H. M. A. Bartels, Erik J. van Lindert, Daniel Hänggi, Hieronymus D. Boogaarts

**Affiliations:** 1 Department of Radiology, Radboud University Medical Center, Nijmegen, The Netherlands; 2 Department of Neurosurgery, Radboud University Medical Center, Geert Grooteplein-Zuid 10, P.O. Box 9101, 6500 HB Nijmegen, The Netherlands; 3 Department of Neurosurgery, Heinrich Heine University Düsseldorf, Düsseldorf, Germany

**Keywords:** Gravitational valve, Adjustable valve, Hydrocephalus, Aneurysmal subarachnoid hemorrhage

## Abstract

**Purpose:**

Hydrocephalus requiring permanent CSF shunting after aneurysmal subarachnoid hemorrhage (aSAH) is frequent. It is unknown which type of valve is optimal. This study evaluates if the revision rate of gravitational differential pressure valves (G-DPVs, GAV® system (B Braun)) (G-DPV) is comparable to adjustable pressure valves (Codman Medos Hakim) (APV) in the treatment of post-aSAH hydrocephalus.

**Methods:**

The use of a gravitational differential pressure valve is placed in direct comparison with an adjustable pressure valve system. A retrospective chart review is performed to compare the revision rates for the two valve systems.

**Results:**

Within the registry from Radboud University Medical Center, 641 patients with a SAH could be identified from 1 January 2013 until 1 January 2019, whereas at the Heinrich Heine University, 617 patients were identified, totaling 1258 patients who suffered from aSAH. At Radboud University Medical Center, a gravitational differential pressure valve is used, whereas at the Heinrich Heine University, an adjustable pressure valve system is used. One hundred sixty-six (13%) patients required permanent ventricular peritoneal or atrial shunting. Shunt dysfunction occurred in 36 patients: 13 patients of the 53 (25%) of the gravitational shunt cohort, and in 23 of the 113 (20%) patients with an adjustable shunt (*p* = 0.54). Revision was performed at a mean time of 3.2 months after implantation with the gravitational system and 8.2 months with the adjustable shunt system. Combined rates of over- and underdrainage leading to revision were 7.5% (4/53) for the gravitational and 3.5% (4/113) for the adjustable valve system (*p* = 0 .27).

**Conclusion:**

The current study does not show a benefit of a gravitational pressure valve (GAV® system) over an adjustable pressure valve (CODMAN ® HAKIM®) in the treatment of post-aSAH hydrocephalus. The overall need for revision is high and warrants further improvements in care.

## Introduction

Gravitational valves are developed to reduce the risk of overdrainage and associated complications, such as subdural effusions in the treatment of hydrocephalus. The use of gravitational valves has been proven beneficial in reducing these complications in different causes of hydrocephalus but especially in idiopathic normal pressure hydrocephalus [8]. Hydrocephalus after aneurysmal subarachnoid hemorrhage (aSAH) is a frequent complication of the disease that occurs in up to 30% of the patients. Next to high- and normal-pressure hydrocephalus, also the gradual change from high- to normal-pressure hydrocephalus in a patient may occur during the course of the disease, which may justify the need for adjustable pressure valves [13].

A recent meta-analysis of two studies investigated the use of fixed differential pressure ventriculoperitoneal shunt valves (DPV) versus adjustable pressure valves (APV) for hydrocephalus following aSAH [7, 13, 17]. It showed that the revision rate was lower in the APV group and a cost–benefit analysis also was in favor of the APV [17]. The Medtronic system was used (Delta versus Strata) in both studies. Although the Delta and Strata system are reported to be gravitational systems, there is only a marginal difference between horizontal and vertical position (about 1.5 cm H_2_0) in contrast to a true gravitational system [11].

This study evaluates if the revision rate of gravitational differential pressure valves (G-DPVs, GAV® system (B Braun)) (G-DPV) is comparable to adjustable pressure valves (Codman Medos Hakim) (APV) in the treatment of post-aSAH hydrocephalus.

## Methods

The STROBE guidelines were followed for the collection and reporting of data [20]. Medical records were retrospectively analyzed for all consecutively treated aSAH patients of 18 years or older who required ventriculoperitoneal or ventriculo-atrial shunt placement between January 2013 and January 2019 at the Neurosurgery Departments of both Radboud University Medical Center in Nijmegen, the Netherlands, and the Heinrich Heine University Dusseldorf, Germany. The G-DPV from the MIETHKE GAV® (B Braun, Hessen, Germany) was used at Radboud University Medical Center. The APV from CODMAN ® HAKIM® (CMH) (Codman; Johnson & Johnson Co., Raynham, MA) was used at the Heinrich Heine University Dusseldorf.

The primary outcome was shunt dysfunction, warranting revision of the system. Secondary outcomes were the occurrence of clinical and neuroradiological overdrainage, shunt obstruction and location of obstruction if present, infection rates (defined by at least one positive cerebrospinal fluid (CSF) culture at microbiological evaluation), ventriculomegaly slit ventricles, and a need for adjustment in the APV group.

Clinical overdrainage was defined as clinical symptoms (headache, nausea) occurring in the upright position with prompt disappearance in the prone position [6]. Radiological overdrainage was defined as the enlargement of the subarachnoid space over the convexity > 3 mm (hygroma) or a subdural hematoma thicker than 2 mm or slit ventricles together with disproportionally wide cortical sulci.

Baseline characteristics were registered: age, sex, American Society of Anesthesiologists (ASA) score, World Federation of Neurosurgical Societies (WFNS) grade, modified Fisher grade, procedural complications, the presence of slit ventricles, antithrombotic medication, and location of the shunt (frontal, temporal, bilateral shunts). Follow-up time was defined as the time in months from drain implantation to the last clinical or radiological follow-up appointment.

The EVD weaning protocol differed at the two centers. At the Radboudumc, weaning was performed by closing the drain for 24 h. If patient condition remained stable and the pressure was below 20 cmH_2_O, the EVD was removed. At the Heinrich Heine University Dusseldorf (HH Dusseldorf), the EVD overflow chamber was raised to 20 cmH_2_O on day 1 and to 25 cmH_2_O on day 2. If the patient remained clinically stable, a CT scan was performed at the end of day 2. Thereafter, the external drain could be removed.

Statistical analyses were performed with SPSS (Software SPSS — version 22, SPSS Inc., Chicago, IL, USA). Statistical significance was assumed when *p* < 0.05. Mean and standard deviations were calculated for continuous, normally distributed variables, while median and interquartiles were provided for continuous, non-normally distributed variables. Frequencies were calculated for all categorical data. Univariate analysis was performed to evaluate differences in baseline. Chi-square or Fisher exact test for all categorical data or independent *t* test was used when appropriate. Normality for continuous data was tested using the Shapiro–Wilk test. To compare two groups if continuous variables were skewed, the Mann–Whitney *U* test was used. A Kaplan–Meier survival analysis was performed for shunt revision for the two different systems.

## Results

**Fig. 1:**
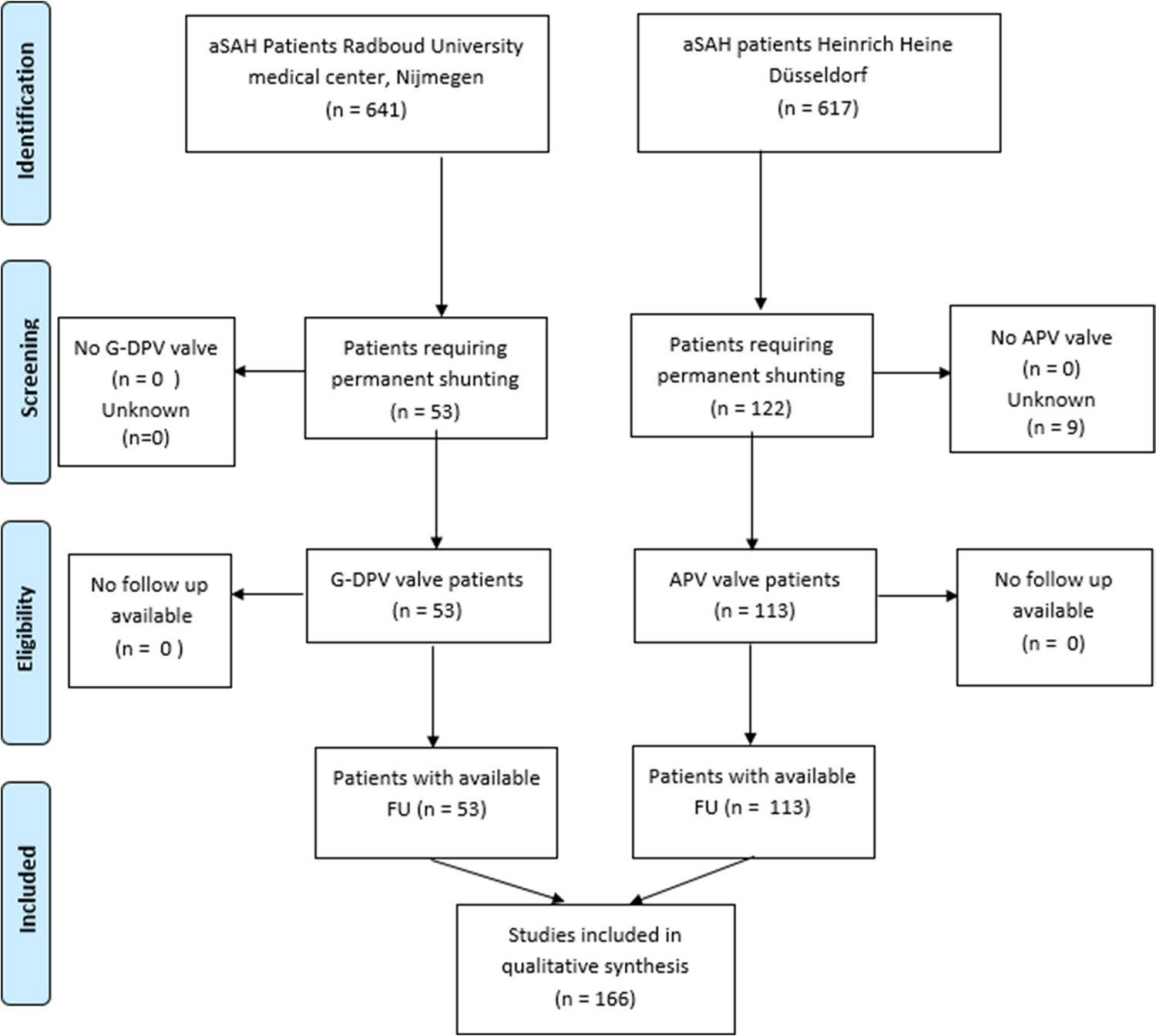
PRISMA flow diagram of included patients

From 1 January 2013 until 1 January 2019, a total of 1258 aSAH patients could be identified; 641 were admitted to Radboud University Medical Center, 617 to Heinrich Heine University. Fifty-three of the 641 aSAH patients (8.2%) at Radboud University Medical Center required permanent ventricular peritoneal or atrial shunting, and this group will be referred to as the G-DPV cohort. At the Heinrich Heine University, 113 of the 617 aSAH patients (18.3%) required permanent ventricular peritoneal or atrial shunting, and this group will be referred to as the APV cohort. The overall combined rate of shunted patients was 13%. In nine cases, the type of shunt was not registered. The selection of patients is shown in Fig. 1. Baseline characteristics are provided in Table 1.

**Table 1:** Demographic data of the shunted patients in both centers

			Radboud University Medical Center		Heinrich Heine University		*p*-value
Number of SAH patients			641		617		n.a
Number of patients needing a permanent CSF shunt (*n*)			53 G-DPV (8.3%)		113 APV (18.3%)		*p* = 0.00
Sex; female (*n*)			32 (60.4%)		79 (70.0%)		*p* = 0.30
Age (mean)			60 (SD = 10)		58 (SD = 12)		*p* = 0.38
WFNS (median)			4 (IQR = 2) (*N* = 52)		4 (IQR = 3) (*N* = 112)		*p* = 0.48
mFisher (median)			4 (IQR = 1) (*N* = 50)		4 (IQR = 1) (*N* = 111)		*p* = 0.15
ASA (median)			2 (IQR = 1)		2 (IQR = 1) (*N* = 95)		*p* = 0.08
Treatment	Endovascular		48 (90.6%)		65 (57.5%)		*p* = 0.00
	• Coiling		34		49		
	• Stent-assisted coiling		8		9		
	• WEB		3		0		
	• Flow diverter		3		7		
	Clipping		4 (7.5%)		47 (41.9%)		*p* = 0.00
	Clip and coil		1 (1.9%)		1 (0.9%)		*p* = 0.54
VPS:VAS			52:1		113:0		
Position ventricular catheter*		Adequate	32	53 (100.0%)	111	111 (98.2%)	*p* = 1.00**
		Touches ependyma	21		0		
		Malposition	0 (0.0%)		2 (1.8%)		*p* = 1.00
Antithrombotic/coagulant medication		Prescribed	13 (24.5%)		44 (39.0%)		*p* = 0.10
		None	40 (75.5%)		15 (13.3%)		*p* = 0.00
		UNK	0 (0.0%)		54 (47.8%)		*p* = 0.00
Median clinical follow-up time from shunt implantation in months			19 (IQR = 32)		8.0 (IQR = 24)		*p* = 0.00
Median radiological follow-up time from shunt implantation in months			17 (IQR = 33)		6.0 (IQR = 33)		*p* = 0.08

*G-DPV* gravitational differential pressure valve, *APV* adjustable pressure valve, *after Hayhurst et al., 2010 [4]; *UNK* unknown, *n.a.* not applicable, *SD* standard deviation, *IQR* interquartile range, *WEB* Woven EndoBridge (WEB; Microvention, Aliso Viejo, CA, USA), *VPS* ventriculoperitoneal shunt, *VAS* ventriculo-atrial shunt. **Combined *p* value of adequate position group and touches ependyma group

**Fig. 2:**
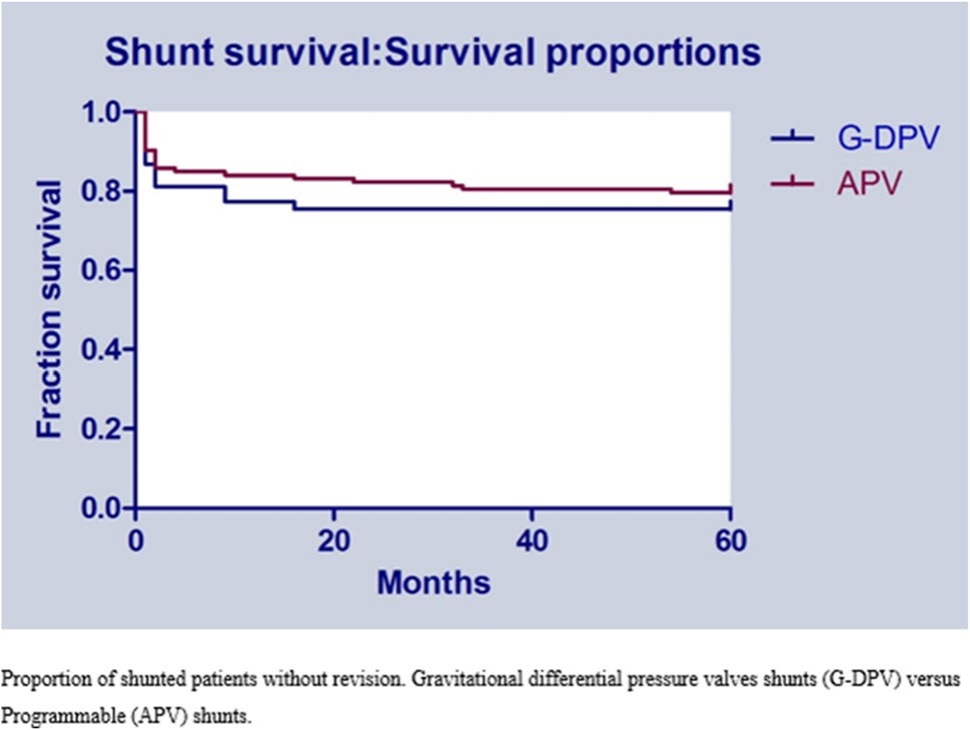
Shunt survival

The median time from aSAH until shunt placement was 27 (range 9–285) days at the Radboudumc and 18 (range 5–707) days at Heinrich Heine University. Shunt revision was needed in 13 patients in the G-DPV cohort (24.5%), and in 23 patients in the APV cohort (20.4%) (*p* = 0.54), resulting in a total revision rate of 21.7%. Shunt revision was performed at a mean time of 3.2 and 8.2 months after implantation at the G-DPV and APV, respectively (*p* = 0.63). The time until revision is graphically depicted in a Kaplan–Meier curve (Fig. 2). Causes of shunt disfunction are provided in Table 2. In the APV cohort, shunt revision was performed by replacing the entire system without investigation of the exact origin of the obstruction in 10 patients. More than one shunt surgery was needed in seven patients for the G-DPV cohort; a revision was needed in three patients, a second revision in one patient, and a third revision in three. Multiple surgeries were performed in seven patients of the APV cohort; a revision was needed in six patients and a second revision was performed in one patient. Combining overdrainage and underdrainage led to a revision rate of 7.5% (4/53) for the G-DPV and 3.5% (4/113) for the APV system (*p* = 0 0.27). One or more valve adjustments in the APV group were required in 46 patients (27 cases of underdrainage, 15 of overdrainage, and 4 missing values). Radiological abnormalities (hygroma, slit ventricles, and ventriculomegaly) were present in 3.8% (2/53) of the G-DPV cohort and 26.5% (30/113) of the APV cohort (*p* = 0.001) but did not require surgical revision. No significant difference was observed between the APV and G-DPV group regarding radiological signs of overdrainage.

**Table 2:** Primary and secondary outcomes

			G-DPV	APV	*p*-value
Revision *n*, %			13 (24.5%)	23 (20.4%)	*p* = 0.54
Reason first revision	Defect valve		4 (7.5%)	0 (0.0%)	*p* = 0.01
	Occlusion (not valve related)		3 (5.7%)	2 (1.8%)	*p* = 0.33
	Overdrainage		1 (1.9%)	0 (0.0%)	*p* = 0.27
	Underdrainage		3 (5.7%)	4 (3.5%)	
	Infection		1 (1.9%)	4 (3.5%)	*p* = 0.63
	Malposition		0 (0.0%)	1 (0.9%)	*p* = 1.00
	No identifiable cause		1 (1.9%)	1 (0.9%)	*p* = 1.00
	Not reported/investigated		0 (0.0%)	10 (8.8%)	*p* = 0.01
	Missing data		0 (0.0%)	1 (0.9%)	*p* = 1.00
Time to first revision (months)			3.2 (SD = 5.0)	8.2 (SD = 14)	*p* = 0.63
Radiological findings *not* requiring surgical revision		Hygroma	2 (3.8%)	10 (8.9%)	*p* = 0.34
		Slit ventricles	0 (0.0%)	6 (5.3%)	*p* = 0.18
		Ventriculomegaly	0 (0.0%)	14 (12.4%)	*p* = 0.01

*SD* standard deviation

## Discussion

The present study reports the use of a true G-DPV compared to an APV in hydrocephalus after aSAH. Overall revision rates were 24.5% and 20.4% for the G-DPV and APV system, respectively. Combining over- and underdrainage, the revision rate was 7.5% and 3.5% for the G-DPV and APV system, respectively. Although the rate of revision for over- and underdrainage seemed to differ, it did not reach statistical significance. The overall revision rate was not different for either system.

The reason for revision in the G-DPV cohort was mainly (13.2%) due to a defect valve or occlusion. In the APV cohort, it was not investigated in a substantial number of patients (8.8%) due to local practice. Therefore, conclusions on the causes of specific malfunction other than overdrainage or underdrainage cannot be made in this cohort of patients.

### Literature reports on shunting after aSAH

Several studies reported on shunt survival in aSAH using a specified valve type [5, 7, 9, 12, 13] Revision rates ranged from 4.2 to 30.3% (Table 3). Overall revision rate in the extant literature combined with our present results was 18.6%. Three studies reporting the use of the Strata system (APV) had revision rates ranging from 7.0 to 21.0% [7, 9, 13, 18]. Of those, two studies compared the use of the Strata valve to the Delta valve at the same location. Both studies had low revision rates (7.0 and 9.1%). It was at the discretion of the surgeon which valve to use, potentially leading to selection bias. One study had limited follow-up for the APV cohort in comparison with the DPV cohort, which might have influenced the results (5.4 versus 24.9 months) [13]. The results of the published APV revision rates were better compared to the revision rates of the APV cohort in the present study. The only marginal gravitational difference of the Strata system was unlikely to be the explanation of the difference with the CMH valve [11]. One other study reported the use of the CMH valve and had a low revision rate of 4.2%, but also had limited follow-up time [12]. It is known that shunt revisions can occur relatively late, i.e., 1 year after implantation [9]. A prospective randomized trial on the use of the CMH found a revision rate of 52% at 2 years, with a mixed variety of underlying pathology [14].

**Table 3:** Literature overview: shunt revision rates in SAH with specified valves

Study, year	Valve	G-DPV	APV	Revision rate % (*n*/*n* total)	FU
Hertel et al. 2008 [5]	Miethke Dual Switch Valve	+	−	21.1% (8/38)	47.6 months mean
Lee et al. 2014 [7]	Strata II	+	+	7.0% (4/57)	26.3 months (both groups) mean
	Delta	+	−	21.6% (8/37)	26.3 months (both groups) mean
Nowak et al. 2018 [12]	CMH with siphonguard	+	+	4.2% (1/24)	3–6 months range
Orrego-Gonzales et al. 2020 [13]	Strata II	+	+	9.1% (3/33)	5.4 months median
	Delta	+	−	30.3% (10/33)	24.9 months median
Mansoor et al. 2021 [9]	Strata*	+	+	21.0% (17/81)	49.9 months median
Radboud, present series	Miethke GAV	+	−	24.5% (13/53)	20 median **
HH Dusseldorf, present series	CMH	−	+	20.4% (23/113)	12 median **
Overall revision rate				18.6% (87/469)	

*CMH* Codman Medos Hakim; *majority (89.8%) of entire group of 227 of which 81 were SAH patients who received Strata valves, *mo* months. **Latest follow-up regardless of radiological or clinical follow-up

Additionally, several other studies investigated revision rates of shunts used in the setting of aSAH but did not specify the type of valve used: One report compared pressure-regulated valves (including valveless, non-adjustable pressure valves, and adjustable pressure valves), flow-regulated valves, and shunts with any valve plus a gravitational unit [19]. Revision rates were 25.0% (5/20), 13.3% (2/15), and 33.3% (4/12), respectively; shunt types were not specified and minimum follow-up was 6 months [19]. Another study reported a revision rate of 42.2% (35/83) at the 6-month follow-up, with no specified valve system [16]. In a recently published study, adjustable valve (25/101), valveless (11/16), and fixed pressure valve (75/232) shunts were compared; however, no distinction was made between adjustable valve shunts with (*n* = 15) and without (*n* = 86) an anti-siphon component regarding revision rate [18]. Overall revision rates in these non-specific studies were variable but high: 32.8% (157/479).

### Shunt dependency after aSAH

The cause of different rates of shunt dependency in the present two cohorts (8.3% and 18.3%) is not clear. Shunt dependency is mostly related to highly modified Fisher grades [21], but these were similar in both groups. Open surgical treatment (a significantly higher percentage in the APV group) is related to a lower non-significant incidence of shunt-dependent hydrocephalus; accordingly, inverse results would be expected [2, 15]. In both centers, the weaning of an external shunt was attempted before deciding for an internal shunt placement, depending on clinical condition in combination with ventriculomegaly or transependymal effusions. Typically, shunt dependency rates after SAH are around 20% [3]. The higher shunt dependency rate could potentially result in a percentage of patients eventually not requiring an internal shunt; a potential dysfunction in these patients would therefore not lead to clinical or radiological abnormalities, lowering the overall risk of revision. Hydrocephalus after SAH is known for its variable occurrence during the early weeks in combination with different pressure characteristics. Commonly, early in the course of the disease, a high-pressure hydrocephalus can occur, whereas a normal-pressure hydrocephalus is seen more often in the later stages. Even changes in the type of hydrocephalus can occur, justifying the need for APV [13]. The presence of acute hydrocephalus requiring shunting within the first 24 h of the disease was not registered in this report; however, baseline characteristics were similar in both groups.

### Limitations

This study has several limitations: First, it is limited by its retrospective nature, relying on chart review and available follow-up. For example, in nine patients of the APV cohort, the type of shunt could not be identified and had to be excluded. Despite being a retrospective case series, it is a large consecutive data set in a non-randomized but internally optimized setting. Although a randomized setting might be optimal from a methodological point of view, it is often not optimal for the setting of shunting due to the experience required for a specific type of valve. Pragmatic registry-based observational studies (PROS) might serve as a future methodal framework to answer systematically the question of the optimal valve type [10]. Secondly, follow-up was only performed when clinically indicated or required in the setting of aneurysm control. However, neurological decline would normally lead to hospitalization at the tertiary care hospital, lessening the chance that making it less likely a shunt malfunction would be missed. Differences in indication for shunting leading to differences in rates of shunted patients could also have influenced the results, as discussed above. Thirdly, because a number of neurosurgeons and residents in training performed surgery, differences in expertise levels might have led to differences in revision rates, although ventricular catheter malposition was rare. Last, since each center implanted one type of valve, surgical techniques and local insertion protocols may be a confounder in this study.

Due to the heterogeneity and the nature of the study designs of published literature, no clear conclusion can be drawn on which type of valve is preferable in the setting of hydrocephalus after aSAH. The overall revision rate is high (18.6%) in the series in which the valve type is specified. Associated readmission rates and associated costs are high in patients suffering from hydrocephalus after aSAH [1]. Therefore, future research on improvements in the treatment of post-aSAH hydrocephalus is greatly needed.

## Conclusion

The current study does not show a benefit of a gravitational pressure valves (GAV® system) over an adjustable pressure valves (CODMAN ® HAKIM®) in the treatment of post-aSAH hydrocephalus. The overall need for revision is high and warrants further improvements in care.
